# Investigating force-time characteristics of prone thoracic SMT and self-reported patient outcome measures: a feasibility study

**DOI:** 10.1186/s12998-023-00491-3

**Published:** 2023-07-07

**Authors:** Grand Choi, Dominic Giuliano, Anthony Tibbles, Samuel J. Howarth, Steve Tran, Joyce Lee, Martha Funabashi

**Affiliations:** 1 Canadian Memorial Chiropractic College, 6100 Leslie St, Toronto, ON M2H 3J1 Canada; 2 Université du Québec à Trois-Rivières, 3351, boulevard des Forges, Trois-Rivières, QC G9A 5H7 Canada

**Keywords:** Spinal manipulative therapy, Thoracic spine pain, Force measurement, Clinical setting

## Abstract

**Background:**

Spinal manipulative therapy (SMT) is commonly used to treat musculoskeletal conditions, including thoracic spine pain. Applying patient-specific force-time characteristics are believed to be important to improve SMT’s effectiveness. Investigating SMT as part of a multimodal approach is fundamental to account for the complexity of chiropractic clinical practice. Therefore, pragmatic investigations balancing minimal disruptions to the clinical encounter at the same time as ensuring a robust data quality with rigorous protocols are needed. Consequently, preliminary studies are required to assess the study protocol, quality of data recorded and the sustainability of such investigation. Therefore, this study examined the feasibility of investigating SMT force-time characteristics and clinical outcome measures in a clinical setting.

**Methods:**

In this mixed-methods study, providers recorded thoracic SMT force-time characteristics delivered to patients with thoracic spinal pain during regular clinical encounters. Self-reported clinical outcomes of pain, stiffness, comfort during the SMT (using an electronic visual analogue scale), and global rating of change scale were measured before and after each SMT application. Feasibility was quantitatively assessed for participant recruitment, data collection and data quality. Qualitative data assessed participants’ perceptions on the impact of data collection on patient management and clinical flow.

**Results:**

Twelve providers (58% female, 27.3 ± 5.0 years old) and twelve patients (58% female, 37.2 ± 14.0 years old) participated in the study. Enrolment rate was greater than 40%, data collection rate was 49% and erroneous data was less than 5%. Participant acceptance was good with both providers and patients reporting positive experience with the study.

**Conclusions:**

Recording SMT force-time characteristics and self-reported clinical outcome measures during a clinical encounter may be feasible with specific modification to the current protocol. The study protocol did not negatively impact patient management. Specific strategies to optimize the data collection protocol for the development of a large clinical database are being developed.

## Background

Cervical, thoracic and lumbar spine related pain are highly prevalent conditions, which collectively are considered to have the largest economic health burdens in western societies [1, 2]. Thoracic spine pain has been described to be as disabling as cervical and lumbar spine pain, having similar incidence with about 45% reporting persistent pain [3–5]. Additionally, thoracic spine pain has been reported to impose major individual and societal burden, significantly contributing to high healthcare-related costs and missed working days [6–8]. Despite its high prevalence and burden, thoracic spine pain has been the focus of considerably less clinical research [9] and little is known about effective interventions for this condition [10].

Spinal manipulative therapy (SMT) is a manual therapy technique commonly used to treat musculoskeletal conditions, including thoracic spine pain, and is recommended by many clinical practice guidelines [11–15]. Application of SMT involves modulating several time-varying characteristics of the force applied by the provider, including preload force, peak thrust force, force duration, and loading rate, to accommodate for the unique clinical presentation of each patient [16]. Specifically, previous studies have emphasized the importance of applying patient-specific force-time characteristics to improve SMT’s effectiveness and minimize potential adverse events [16–18]. The therapeutic effects of SMT are believed to be associated with the neuromechanical responses observed following SMT applications. Previous studies have described that different SMT force-time characteristics influence varying physiological responses, including electromyographic and muscle spindle responses [19–24] as well as vertebral displacement and acceleration [25–28]. Specifically, higher peak thrust force magnitudes have been observed to elicit greater muscular response amplitude, muscle spindle discharge and vertebral displacement [19, 25, 29, 30]. Similarly, decreased thrust duration has been reported to increase changes in paraspinal muscle spindle responses [23, 31]. Despite these findings, the potential association between SMT force-time characteristics and patient clinical outcomes remains unclear.

A recent pioneering observational study found that no specific SMT force-time characteristic was associated with short-term clinical responses related to pain, disability and global perceived change [32]. While this study followed a rigorous design specifically focused on SMT, the applications of its findings to a real-world clinical scenario is limited as SMT is often applied as part of a management plan that includes other interventions, such as soft tissue therapy and exercise. The authors also highlighted their study’s short-term follow-up, which limits the application of their findings to a real-world clinical scenario, where patients usually receive more than one SMT and are treated for longer than 7 days by their chiropractors.

While the focus on SMT force-time characteristics in isolation from other therapies allow for the elucidation of SMT-specific effects, investigating SMT as part of a multimodal approach is fundamental to account for the complexity of current chiropractic clinical practice. Therefore, a pragmatic approach in which SMT is part of a management plan that includes other interventions is fundamental to better understand the real-world effects that are observed in clinical practice. Particularly to the thoracic spine, one common SMT technique consists of patients lying prone and the clinician applying a posterior-to-anterior force to the thoracic spine. Compared to other SMT techniques that usually combine movements of flexion or extension, lateral bending and rotation of the spine in addition to the force application, thoracic SMT in prone position is ideally suited to quantify SMT forces in a clinical setting without interfering with the flow of the clinical encounter.

If pragmatic clinical investigations recording SMT force-time characteristics were conducted without disrupting clinical encounters, then data could not only be collected for a longer period of time, which would better reflect real-world patient management, but could also potentially be integrated as a regular part of clinical practice. This way, a growing database could be developed with robust data related to SMT force-time characteristics and patient clinical outcomes, best reflecting the real-world clinical scenario. Combined with other clinically relevant factors (e.g., patients’ conditions, individual characteristics, expectations, and preferences), this database could contribute to the identification of potential associations between SMT force-time characteristics and patient clinical outcomes. If such associations exist, identifying them could lead to important advances not only scientifically by focusing mechanistic investigations on the specific SMT force-time characteristics that influence patient outcomes, but also clinically by training students and informing clinicians on the SMT characteristics that most influence clinical outcomes, enabling them to provide a more evidence-based therapeutic approach.

Such studies, however, are very difficult to develop as they require a fine balance between minimizing disruptions to clinical encounters at the same time as ensuring a robust data quality with rigorous protocols. Consequently, preliminary studies are needed to fully assess the developed protocol, quality of data recorded, perceptions of the study protocol from both patients and providers, and its sustainability to be integrated into the clinical routine. Therefore, the primary purpose of this study was to explore the feasibility of collecting SMT force-time characteristics and clinical outcome measures in a clinical setting. The secondary aim of this study was to determine the variability in SMT force-time characteristic values and patient self-reported outcome measures in those with thoracic spine pain.

## Methods

### Study design

A sequential explanatory mixed-methods observational study design was used to explore the feasibility of recording SMT force-time characteristics and self-reported patient outcome measures in a clinical setting. Quantitative data were collected to objectively investigate the primary outcomes of feasibility including data quality, data collection rates, and participant recruitment and retention rates. Qualitative data were collected to assess the perceptions and attitudes of both providers and patients on perceived impact of data collection on patient management and clinical flow. Qualitative data helped to explain the quantitative data by providing context to the feasibility outcomes observed.

Although this study was originally planned to be conducted between February 2020 and May 2020, data collection ceased early due to the COVID-19 pandemic and the closure of the clinic in mid-March 2020. Therefore, data collection occurred between February and mid-March 2020.

### Population

A convenience sample of providers and patients from the Canadian Memorial Chiropractic College (CMCC) campus clinic were invited to participate in the study. Providers included student interns and their supervising clinician. Inclusion criteria included providers treating at the CMCC campus clinic and providing prone thoracic SMT procedures. Providers were excluded if they had any injuries or conditions that would prevent them from safely performing SMT procedures. Patients were included if they were over 18 years of age, fluent in English, had a current diagnosis of mechanical thoracic spine pain, and receiving prone thoracic SMT as part of their plan of management. Patients were excluded if they had any history of spinal surgery, or if their thoracic spine symptoms were likely attributable to non-musculoskeletal causes, such as inflammatory arthritis, neoplasms, infections, or visceral referred pain.

### Recruitment

This study was reviewed and approved by the CMCC Research Ethics Board (REB approval # 1905B02). All participants (providers and patients) signed a written informed consent prior to participating in the study.

Providers were informed about the study via e-mail and in-person visits by a study team member (GC) to the providers’ pod to explain the study. Once enrolled, all providers were trained by one of the investigators through presentations detailing the study protocol and procedures as well as simulated study visits.

Based on their electronic health records, potentially eligible patients were identified based on their primary condition for receiving treatment and checked against the study’s inclusion and exclusion criteria, with eligible patients being invited to participate in-person by a study team member (GC/JL). Self-reported demographic and anthropometric data were collected from all participants (providers and patients) using an online survey immediately after obtaining informed consent.

### Study protocol

Participating providers were instructed to begin their patient encounter as they normally would by taking a brief subjective history. After this, patients were provided with a tablet by the provider to complete the electronic pre-SMT outcome measures (detailed in *Instrumentation* section). Patients were instructed not to share their submissions with the providers to avoid influencing the provider’s response and the patient-provider relationship.

Providers then continued with the physical evaluation which included an assessment to determine if a thoracic SMT was clinically indicated and, if it was, the SMT application site. After the physical evaluation, participating providers delivered all thoracic SMT in prone position, if clinically indicated, before other interventions. Participating providers were instructed to initiate the recording of SMT force-time data prior to each application of thoracic SMT procedures, including when multiple thoracic SMT procedures were applied to the same patient at the same visit.

After the delivery of all prone SMT procedures, patients were asked to complete their post-SMT outcome measures (detailed in *Instrumentation* section). Providers were also asked to complete their online survey (detailed below) on a separate tablet. Following this, interns continued with the rest of the clinical interaction as they normally would. This process was repeated in every visit each participating patient had during the study period, if possible.

After the data collection period ended, qualitative follow-up surveys regarding attitudes and experiences were e-mailed to all participating providers and patients.

### Instrumentation

The online surveys were specifically developed for this study using the SurveyMonkey electronic data capture platform (Nomentive Inc., San Mateo, California, USA; www.surveymonkey.com). At each visit, participating patients were asked to complete two surveys: one prior to the thoracic SMT procedures and another immediately after SMT. The pre-SMT survey included separate electronic visual analogue scales (eVAS) for patient’s self-reported current thoracic pain and stiffness where 0 corresponded to no pain/stiffness and 100 corresponded to worst imaginable pain/stiffness. It also included the global rating of change scales (GRoC; ranging from 0 = very much worsened to 7 = very much improved) that measured overall improvement or worsening since study enrolment (except for first visit, where this was not applicable). The post-SMT survey also included the eVASs, GRoC related to their thoracic spine pain, stiffness, overall improvement, and comfort during the thoracic SMT procedures (with 0 = most comfortable and 100 = most uncomfortable). Participating providers were also asked to complete a survey immediately after the thoracic SMT procedures, which included information about the performed SMT procedures, including the levels of SMT application and number and type of prone thoracic procedure. At the end of the study, qualitative surveys with open-ended questions regarding participants’ attitudes and experiences in the study were e-mailed to all participants. Specifically, providers’ qualitative survey included questions regarding their perceptions related to the impact the study had on the clinical flow and effectiveness of care, potential modifications or adaptations to their SMT application due to the FSTT®, their thoughts related to having a longer study and having this protocol being part of their normal clinical routine, to describe any positive or negative experiences they had during the study and if they had any suggestions to improve the study protocol. Patients’ qualitative survey included questions related to their perceptions of the study protocol interfering with their treatment, their overall experience with the pre- and post-SMT surveys as well as with the FSTT®, if they would be open to participating in a longer study or having the protocol as being part of the regular clinical routine, their overall satisfaction and if they had any suggestions to improve the study protocol.

Three-dimensional SMT forces at the patient–table interface were measured and recorded using the Force Sensing Table Technology - FSTT® (Canadian Memorial Chiropractic College, Toronto, ON, Canada) with a sampling rate of 1000 Hz. The FSTT® comprised a treatment table with an embedded force plate (OR6-7; Advanced Mechanical Technology Inc, Watertown, MA, USA). The thoracic portion of the treatment table (with embedded force plate) was mechanically independent from the remainder of the treatment plinth, ensuring that the force plate only captured the interaction between the patients’ thoracic region and the thoracic portion of the treatment table. The FSTT® readings were zeroed after patient positioning and prior to SMT application to account for the patient’s weight. Previous research has demonstrated excellent reliability and validity of the FSTT® in measuring SMT force-time characteristics [16].

### Outcome measures

The primary outcomes of this study were measures of feasibility including: (1) provider and patient recruitment, (2) data collection, (3) resource, (4) data quality, and (5) provider and patient acceptance [33]. Specifically, provider and patient recruitment outcomes included the number of eligible participants, enrolment rate (percentage of eligible participants who enrolled in the study; successful if ≥ 30%), dropout rate (percentage of enrolled participants who dropped out of the study; successful if ≤ 20%), and participation rate (percentage of enrolled participants that underwent data collection and were included in the analysis; successful if ≥ 70%) [34–36]. Data collection outcomes included the number of possible data collection points (count of appointments where data collection should have occurred), data collection rate (percentage of possible data collections that actually occurred; successful if ≥ 70%) and missed data collection rate (percentage of possible data collections missed; successful if ≤ 20%) [34, 36]. Overlap rate (percentage of data collections missed due to overlapping appointment schedules; successful if ≤ 20%) was the resource outcome. Data quality outcomes included rates of incorrect data entries (percentage of entries that were erroneous), data duplication rate (percentage entries that were duplicates), data misnaming rates (percentage of files that had errors with naming or other similar issues), FSTT® analysis errors rate (percentage of files with incorrect automated analysis), missing data rate (percentage of files/entries missing compared to expected number of data collections), and rates of overwritten files (percentage of files that were overwritten, identified as different files presenting the exact same data, but with different dates). All data quality outcomes were considered successful if ≤ 10%. Provider and patient acceptance outcomes included open-ended survey questions and 5-point Likert scales (completely disagree, somewhat disagree, neither agree/disagree, somewhat agree, completely agree) about their expectations and experiences prior to and during the study. Survey questions asked participating providers and patients about their overall satisfaction with participation, impact of study participation on treatment, perceived difference compared to regular appointments, perceptions of loss of appointment time, ease of completion of outcomes/surveys, and attitudes towards longer periods of data collection [36].

**Fig. 1:**
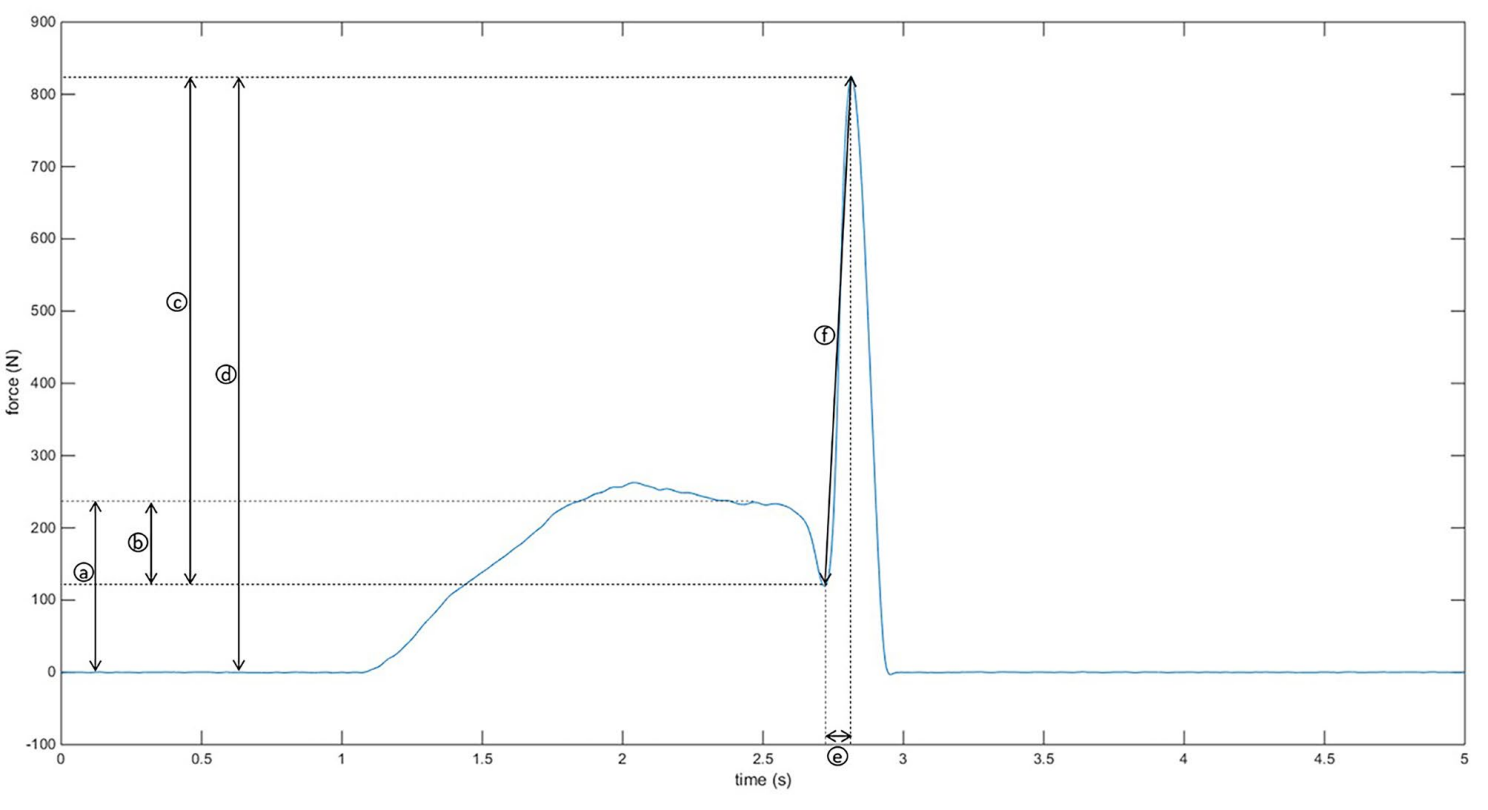
Spinal manipulative therapy force-time characteristics: **a**) preload force; **b**) force dip; **c**) peak thrust force; **d**) total peak force; **e**) time to peak; **f**) loading rate

Secondary outcome measures included SMT force-time characteristics (preload force, force dip, peak thrust force, total peak force, time to peak, and loading rate (Fig. 1) [10, 37]) and clinical outcomes (patients’ self-reported pain, stiffness, comfort level and global rating of change).

These data were recorded by a dedicated computer using a modified FSTT® software that allowed for automated saving of SMT force-time characteristic data. All force variables were recorded along all three axes of the force plate’s reference frame: x-axis = lateral forces relative to the table; y-axis = cephalad/caudal forces; and z-axis = forces perpendicular to the table. The FSTT® software was modified to not visually display any SMT force-time characteristics feedback to avoid influencing provider behavior or performance. Participating providers also did not have access to the FSTT® recorded data.

### Data analysis

Feasibility measures of recruitment, data collection, resource, and data quality were analyzed using counts, frequency, and percentages. Provider and patient acceptance were assessed qualitatively and responses to open-ended questions were coded by one reviewer and a second reviewer checked the codes and themes.

Secondary analysis of the FSTT® data and self-reported outcome measures were conducted to determine the variance in the data to help inform power and sample size calculations for future studies. Raw force plate data were automatically analyzed by the modified FSTT® software with standardized algorithms to identify SMT force-time characteristics. All FSTT® data were manually reviewed to ensure that SMT force-time characteristics were correctly identified by the software. When the automated analysis failed (e.g., failure to detect proper preload force, force dip, and peak force points), relevant points of the force-time graph were manually identified by the principal investigator within the FSTT® software and re-calculated. Median and interquartile range (IQR) were calculated for SMT force-time characteristics. Similarly, median, IQR and change (post-treatment score – pre-treatment score) were calculated for self-reported outcome measure scores. Provider post-SMT online surveys were matched to FSTT® data using the timestamps of the saved files and survey submissions.

## Results

### Participants

**Fig. 2:**
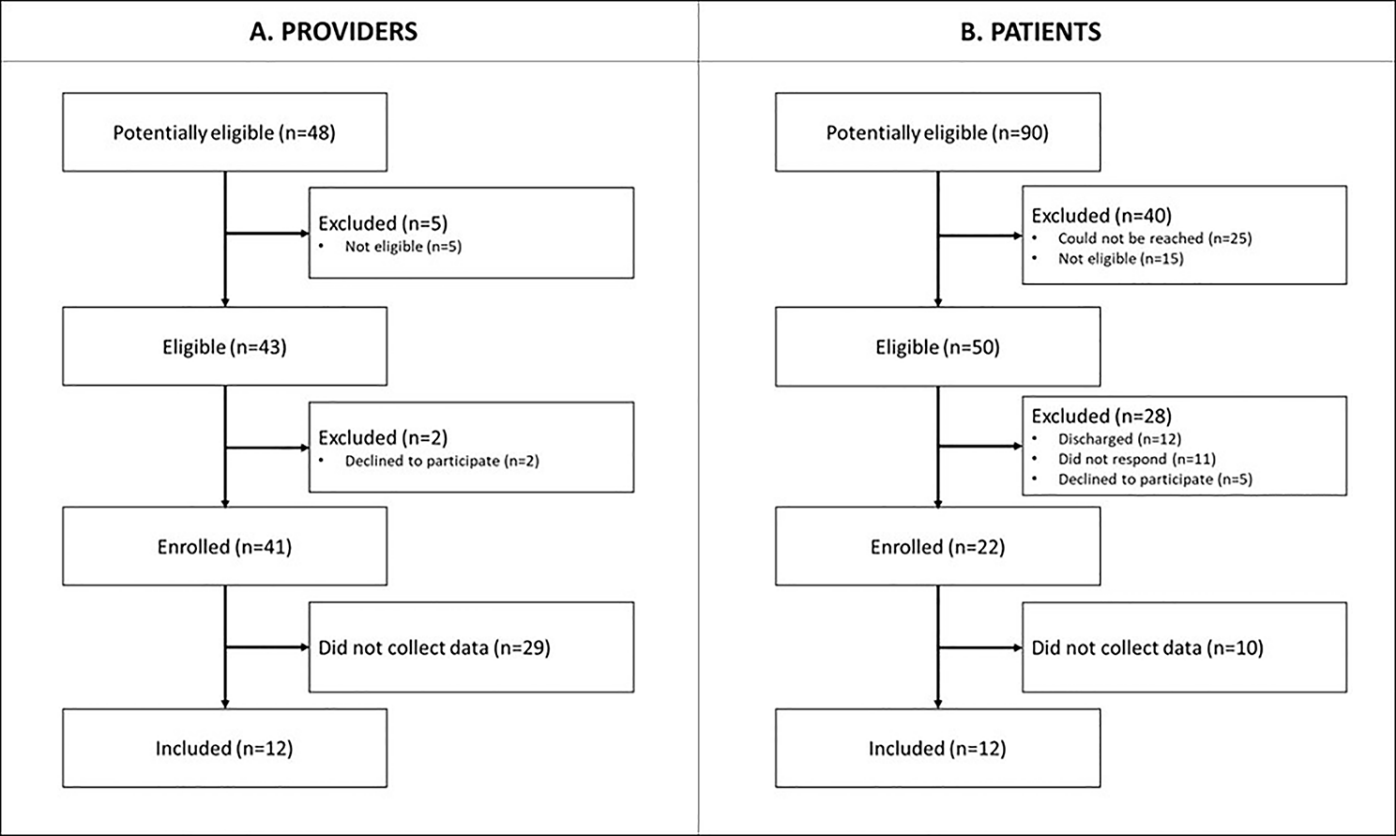
Participant flow chart for: **A** = providers and **B** = patients

Forty-eight providers were potentially eligible to participate. Of these, 5 were excluded as they did not perform prone thoracic SMT procedures. The remaining 43 were invited to participate and of these, 2 declined to participate due to “anxiousness”. The remaining 41 enrolled in the study, however only 12 providers contributed to data collection during the study period and were included in the analysis (Fig. 2A).

Ninety potentially eligible patients were identified: 25 of these could not be reached for invitation to participate in the study, 15 were excluded as they did not receive SMT as part of their treatment. Among the 50 patients who were invited to participate in the study, 17 did not enroll (5 declined; and 12 were being discharged), 11 did not respond to the invitation, and 22 enrolled in the study. Among those who enrolled, 12 participants participated in data collection and were included in the analysis (Fig. 2B). Characteristics of participating providers and patients are shown in Table 1.

**Table 1:** Participant characteristics. Mean (standard deviation [SD]) shown, unless otherwise stated

Characteristic	Provider (n = 12)	Patient (n = 12)
Sex, Female, n (%)	7 (58.3)	7 (58.3)
Age (years)	27.3 (5.0)	37.2 (14.2)
Height (cm)	168.6 (7.4)	168.3 (12.5)
Weight (kg)	67.3 (12.1)	70.9 (17.3)
BMI (kg/m2)	23.6 (3.5)	24.7 (4.0)
Activity level (minutes per week)	228.3 (98.1)	161.0 (193.0)
Previous SMT Exposure, n (%)		
*Cervical Spine*	12 (100)	12 (100)
*Thoracic Spine*	12 (100)	10 (83.3)
*Lumbar Spine*	12 (100)	11 (91.6)
*Pelvis*	12 (100)	8 (66.6)

BMI = Body mass index; SMT = spinal manipulative therapy

Table 2 presents the number of SMT procedures that were applied to each participating patient in each visit and had SMT force-time characteristics data recorded.

**Table 2:** Number of thoracic spinal manipulations with recorded force-time characteristics data per visit for each participating patient

	Data collection visit 1	Data collection visit 2	Data collection visit 3	Data collection visit 4	TOTAL
Participant 1	1	2			**3**
Participant 2	2	2			**4**
Participant 3	3	3	3		**9**
Participant 4	2	3			**5**
Participant 5	1	2			**3**
Participant 6	2	2	3	3	**10**
Participant 7	2	2			**4**
Participant 8	2	3			**5**
Participant 9	2	1	1		**4**
Participant 10	1				**1**
Participant 11	1				**1**
Participant 12	3				**3**
**TOTAL**	**22**	**20**	**7**	**3**	**52**

### Feasibility – provider and patient recruitment, data collection, and resource

Of the 43 eligible providers, 41 enrolled, resulting in a 95.3% enrolment rate. Of these, 12 participants underwent data collection and analysis, resulting in a participation rate of 29.3%. Of the 50 eligible patients invited 22 enrolled resulting in an enrolment rate of 44.0%. Of those enrolled, 12 participants underwent data collection and analysis, resulting in a participation rate of 54.5%. There were no dropouts in both provider and patient groups.

During the data collection period, there were 51 appointments in which data could have been collected. Of these, data collections occurred in 25 appointments, resulting in a collection rate of 49% (51% missed). Of the possible data collections, 4 collections were missed due to overlapping appointments with another participant, resulting in an appointment overlap rate of 7.8%. The remaining 43.2% missed data collections were due to participating provider forgetting to collect data.

### Feasibility – data quality

The rate of incorrect study ID entries among all participants and data collections was 5.1%. Data duplication occurred at a rate of 1.2% and occurred only with surveys. The time stamp data of saved FSTT® files and surveys was incorrect in 28% of the data and was identified as being due to daylights savings time changes and online survey settings. FSTT® auto analysis was incorrect 17.2% of the time and was attributed to signal-to-noise ratio of force-time characteristics and successive manipulation procedures within the same file. Missed data entry rates were 0.6% and overwrite rates were 0.6%.

Missed data and overwritten data were irrecoverable. The remaining errors could be corrected by cross-referencing between the appointment schedule and study ID. All errors were corrected prior to the analysis.

### Feasibility – provider and patient acceptance

Eleven providers responded to the open-ended survey about their expectations and experiences prior to and during the study. Prior to the study, most participating providers believed the study protocol would impact their normal patient interaction. Specifically, time required for data collection was a major concern to all respondents and having to perform SMT first was a concern for 36.4% of provider respondents: “*I typically do adjustments at the end of treatment and was worried they would be sensitive if done at the start of the treatment*” (P007). Some respondents (27.3%) reported they had to be mindful of time, but that it was not significant: “*It takes extra time for the appointment so it was something I had to be mindful of*” (P005). Although most providers reported no need to modify their thoracic SMT technique, one participant reported that the table was not the correct height for them and another felt the table’s cushioning was firmer: “*the table was harder so less comfortable for patients, a little less force*” (P010). While most providers felt that data collection did not impact the effectiveness of their care, one provider felt it impacted their normal rapport with their patient: “*[the study] made it less a personable experience with the patient. We normally chat and catch up on their week at the beginning of the appointment but the protocol interrupted that with the time allotted*.” (P002).

Provider feedback regarding the FSTT® included recommendations to make the table’s height adjustable, cushions less firm, and providing armrests for the patient. They also recommended that multiple FSTT® to be available for data collection due to overlapping of patients who were scheduled at the same time.

Although most providers (63.6%) reported that it would be acceptable and seamless to incorporate the data collection protocol as part of the regular encounter: “*The data collection did not take long at all and my patients were happy to contribute*” (P003), one provider didn’t see the value of making it a regular part of the visit and another was concerned with the impact on patient-centred care. Overall, providers reported having a positive experience as they were able to participate in research, however, it added time to the encounter.

Four participating patients responded to the open-ended survey and reported feeling overall somewhat satisfied (n = 2; 50%) or very satisfied (n = 2; 50%) with participating in the study. Respondents neither agreed or disagreed (50%), somewhat disagreed (25%), or completely disagreed (25%) that the study impacted their treatment. Perceptions regarding appointment time being lost due to data collection varied: one participant somewhat agreed, one neither disagreed or agreed, one somewhat disagreed and one completely disagreed.

Overall, participating patients reported that completing the pre- and post-treatment surveys was easy and not time consuming. They also felt that it did not impact their treatment negatively and that the FSTT® was not noticeably different or more uncomfortable than a regular treatment table. They also expressed openness to a longer data collection period and making this protocol a regular part of the encounter: “*I think it would be a good thing overall*” (P103).

### Force-time characteristics

Overall, most SMT procedures were applied using a bilateral hypothenar technique to thoracic levels between T1 and T8. Within the same treatment visit, two thoracic SMT procedures were mostly applied, with each SMT being applied at different thoracic levels.

Force-time characteristics of posterior-to-anterior thoracic SMT procedures along the three axes of motion are presented in Table 3 and a large variability was observed across all characteristics.

**Table 3:** Force-time characteristics of posterior-to-anterior thoracic spinal manipulative therapy applied during the study

		Fx	Fy	Fz	Fres
**Preload force (N)**	Median [IQR]	7.5 [14.8]	21.0 [29.3]	221.0 [85.3]	223.0 [85.5]
	Min-Max	-15.0–66.0	-36.0–75.0	46.0–368.0	48.0–375.0
**Force dip (N)**	Median [IQR]	1.0 [6.0]	1.5 [5.8]	13.5 [45.5]	13.5 [43.5]
	Min-Max	-5.0–40.0	-5.0–37.0	0.00–145.0	0.00–134.0
**Peak thrust force (N)**	Median [IQR]	16.5 [23.5]	42.5 [36.3]	321.0 [156.3]	325.0 [150.5]
	Min-Max	-39.0–93.0	-40.0–115.0	58.0–636.0	64.0–645.0
**Total peak force (N)**	Median [IQR]	26.0 [33.0]	52.5 [66.0]	526.5 [236.3]	537.0 [226.0]
	Min-Max	-39.0–130.0	-63.0–139.0	122.0–809.0	130.0–817.0
**Time to peak (ms)**	Median [IQR]	133.0 [70.0]	133.0 [43.3]	124.0 [15.3]	124.0 [16.0]
	Min-Max	48.0–573.0	73.0–1101.0	70.0–427.0	76.0–428.0
**Loading rate (N/s)**	Median [IQR]	116.0 [160.5]	312.5 [269.3]	2435.0 [1146.0]	2437.0 [1175.8]
	Min-Max	-260.0–769.0	-416.0–952.0	836.0–5260.0	854.0–5328.0

Fx = medio-lateral forces; Fy = cephalad/caudal forces; Fz = forces perpendicular to the table surface; Fres = resultant vector magnitude; IQR = interquartile range; N = Newtons; ms = milliseconds; N/s = Newtons per second

### Patient self-reported outcome measures

Change in self-reported pain and stiffness between pre-SMT and post-SMT were highly variable. Thoracic SMT-related comfort/discomfort was also highly variable (Table 4).

**Table 4:** Patient self-reported scores for pain, stiffness and comfort during thoracic spinal manipulative therapy

Measure		
**Change in pain**	Median [IQR]	-8.5 [-31.2]
	Min-Max	-50–6
**Change in stiffness**	Median [IQR]	-11.5 [-26]
	Min-Max	-50–0
**SMT-related Comfort / Discomfort**	Median [IQR]	10 [21]
	Min-Max	0–60

Among the 25 appointments where data collection occurred, none of the participants reported worsening of their thoracic spine complaint. No significant change compared to the pre-SMT state was reported in 14 of the sessions (minimally worsened, n = 0; no change, n = 6; minimally improved, n = 8). Overall improvement was reported in 11 sessions (much improved, n = 9; very much improved, n = 2).

## Discussion

This is the first study to record SMT force-time characteristics during regular clinical encounters, pioneering the investigations of the potential associations between SMT force-time characteristics and clinical outcomes within a real-life clinical multimodal approach. Results indicate participant recruitment and data quality may be acceptable. Participant acceptance was good with both providers and patients reporting positive experiences with the study and being open to incorporate the data collection protocol as part of the clinical encounter. Secondary outcomes suggest large variability in the SMT force-time characteristics and self-reported clinical outcomes. This study showed that it may be feasible to record SMT force-time characteristics and clinical outcome measures during a clinical encounter, and identified specific barriers and facilitators to improve the protocol of future larger studies.

While enrolment rates of both providers and patients were acceptable, participation rates were lower than expected, with only 12 providers and 12 patients participating in the study. This was greatly related to the COVID-19 pandemic resulting in reduced clinic visitations and its closure. Consequently, the study was significantly shortened restricting the period for data collection. As such, the actual participation rates and data collection in this study may differ from normal clinic operations and future studies should take this into consideration. Regardless of that, enhanced study protocol strategies were developed to address the 43% missed data collections due to participating providers forgetting to collect data. Specifically, no data were collected in all missed data collection occasions (i.e., there were no data partially collected). Therefore, strategies developed to address this challenge include flagging participating patients in the system and sending weekly study reminders to participating providers. These were developed to minimize the cognitive challenges associated with research activities added to the already cognitively demanding clinical encounters.

Data quality overall was considered good with very few irrecoverable data errors. Despite the identified potential sources of error, most errors could be corrected, with only 1.2% being non-correctable errors. Importantly, reasons for data errors were identified and specific mitigation strategies are being developed to minimize data errors in future studies, such as using more robust data collection instruments (e.g., alternative survey platforms) and procedures (e.g., accounting for daylight saving time change and study ID reminder strategies).

While providers reported being concerned with the data collection protocol negatively impacting patient management, the same was not shared by patients. In fact, patients reported that the study protocol did not interfere with their care and were open to the idea of making such data collection a regular part of the encounter. Future studies can ease providers’ concerns by incorporating patients’ experience regarding their participation in this feasibility study in educational and training sessions. This, in turn, can potentially further improve provider enrolment and participation in future larger studies. It is, however, important to highlight that this study had a limited number of participant qualitative survey responses. Therefore, the reported perceptions related to the study protocol are reflections of the participating providers and patients who responded to the qualitative survey only and may not be generalizable to all participants.

Based on this study’s findings, collecting SMT force-time characteristics during clinical encounters may be feasible. This suggests that the data collection protocol used in this study could be potentially integrated to the clinical practice routine with some modifications to develop a growing database of SMT force-time characteristics and clinical outcome measures. This database, in turn, could significantly contribute to exploring the potential association between SMT force-time characteristics and patient clinical outcomes while also considering the additional complexities of the real-world chiropractic patient management. Nevertheless, even though participants reported being open to longer studies necessary for such a database, the feasibility of longer data collection periods needs to be further assessed.

Pasquier et al. (2022) [32] explored potential short-term prognostic factors for clinically significant changes in pain, disability and global perceived change following SMT in a similar population to our study. Although they found no associations between SMT force-time characteristics and clinical responses, only one SMT application was provided and clinical outcomes were only measured in the short-term (7 days) [32]. Therefore, investigations with a more pragmatic approach, where both SMT delivery and clinical outcomes are continuously delivered and measured (respectively), would better capture the real-world clinical scenario in which SMT is used to treat patients. This is further supported by some of our results where 50% of the encounters reported no improvement immediately after a single application of SMT.

The high variability in SMT force-time characteristics observed in the current study is consistent with previous studies reporting SMT force-time characteristics applied by clinical chiropractors [38]. While the current study observed greater preload and peak force magnitudes, force measurement instrumentation used in each study can partially explain this difference. Specifically, previous studies measured SMT force-time characteristics using pressure pads at the clinician-patient interface, whereas our study used the FSTT®, which measured forces at the patient-table interface. Previous studies reported the differences in SMT force-time characteristics measured at both interfaces [39–41] and provides a potential explanation for greater force magnitudes observed in this study. Another potential reason might be that, in previous studies, SMT was applied to asymptomatic volunteers, whereas in the current study, SMT was applied to patients with thoracic spine pain. This may suggest that the clinical presentation of people receiving SMT may influence the characteristics of the applied SMT. While there are many additional potential reasons for the observed variability in SMT force-time characteristics (such as patient and clinician preferences, table height, etc.), recording SMT forces in clinical settings remains under-investigated and factors influencing this variability are not completely understood yet. Therefore, this study also can also contribute to advance this area and future studies should be conducted to further investigate what factors influence SMT force-time characteristics in clinical settings.

The high variability in patients’ self-reported clinical measures of pre- and post-SMT pain observed in this study is also comparable to the ones reported in previous studies investigating thoracic SMT [32, 42, 43]. While self-perceived stiffness has not been studied following thoracic SMT, a previous study reported that patients consider pain and stiffness as different constructs and that stiffness can also impact their function and activities [44]. Additionally, it has also been reported that subjective or self-reported stiffness is not correlated with objective measures of stiffness [45, 46] and that, similar to pain, perceived stiffness is likely multifactorial, and should be considered in future clinical studies. Importantly, the results of the current study can be used to inform power and sample size calculations of future studies with similar methodology involving patients with thoracic spine pain and prone thoracic SMT.

### Summary of protocol changes

Based on the findings of this study, specific strategies were developed to enhance the study protocol in future studies. To improve participation, a summary of participating providers and patients’ experiences participating in the study could assist with easing potential concerns with study protocol and participation. Strategies to enhance study protocol integration into clinical dynamics and preventing missed data collections include: (1) flagging participating patients in the clinic scheduling system, (2) sending study reminders to participating providers, (3) using more robust data collection instruments (survey platforms) and procedures (with automatic daylight saving time change and study ID reminders), (4) having multiple FSTT® available for data collection, and (5) potential FSTT® design modifications to increase patient comfort and provider preferences.

### Strengths/limitations

The current study has several strengths. Firstly, this study used sequential explanatory mixed-methods approach where quantitative data was used to objectively measure aspects of feasibility and was further supported by qualitative data to provide deeper understanding of participants’ perceptions of the study protocol. Importantly, not only participating clinicians and interns, but also patients contributed to the qualitative data, providing a global understanding of all involved in the study. Additionally, participants were blinded to each other’s responses, which potentially reduced the risk of altered performance or negative consequences to patient-provider relationships.

The largest limitation of this study was the small sample included in this study, likely related to the short data collection period due to the COVID-19 pandemic and clinic closure. This limited the data collection period by 2 months, which possibly influenced in our measures of feasibility. This also caused a delay in gathering information about participants’ experiences due to needing to contact them via e-mail, which required additional ethics approval. This study was also conducted in a teaching clinic. Therefore, the applicability of our results to community-based clinical practices may be limited and further considerations may be needed if similar study is to be conducted in such setting. Finally, low response rate to participant acceptance survey, and lack of established outcome measures for the thoracic spine region may also limit the generalizability of our results.

## Conclusion

Recording good quality SMT force-time characteristics data and self-reported outcome measures during a clinical encounter may be feasible in the short-term (2 months) using the current protocol. Although patients did not feel the study protocol negatively impacted their management, providers raised some concerns including time and flow of encounter. A high degree of variability was observed among both SMT force-time characteristics and self-reported patient outcome measures. Specific strategies to optimize the data collection protocol for the development of a large clinical database are being developed. Future studies should consider the barriers and concerns identified in this study.

## Data Availability

Data from the current study are not publicly available due to reasons of sensitivity surrounding individual human data but are available upon reasonable request to the corresponding author. Data are being stored under controlled access at CMCC.

## Abbreviations

CMCC: Canadian Memorial Chiropractic College
eVAS: Electronic Visual Analogue Scale
FSTT®: Force Sensing Table Technology
GRoC: Global Rating of Change
IQR: Interquartile Range
ms: milliseconds
N: Newtons
N/S: Newtons per second
REB: Research Ethics Board
SD: Standard Deviation
SMT: Spinal manipulative therapy
